# Heterogeneous uptake of ^18^F-FDG and ^18^F-PSMA-1007 PET/CT in lung cancer and lymph node metastasis

**DOI:** 10.1186/s12890-023-02377-9

**Published:** 2023-03-07

**Authors:** Yuan Hu, Peng Wang, Wenli Dai

**Affiliations:** Department of Nuclear Medicine, The First College of Clinical Medical Science, China Three Gorges University, Yichang, 443003 Hubei China

**Keywords:** ^18^F-PSMA-1007, ^18^F-FDG, PET/CT, Primary lung cancer, Lymph node metastases

## Abstract

**Background:**

PSMA PET/CT has shown excellent results in imaging of prostate cancer. However, some nonprostatic malignancies can also demonstrate ^18^ F-PSMA uptake, including primary lung cancer. ^18^ F-FDG PET/CT is widely employed in initial staging, response to therapy and follow-up assessment for lung cancer. Here we present an interesting case report on the different patterns of PSMA and FDG uptake between primary lung cancer and metastatic intrathoracic lymph node metastases in a patient with concurrent metastatic prostate cancer.

**Case presentation:**

A 70-year-old male underwent ^18^ F-FDG PET/CT and ^18^ F-PSMA-1007 PET/CT imaging due to suspicion primary lung cancer and prostate cancer. The patient eventually was diagnosed with non-small cell lung cancer (NSCLC) with mediastinal lymph node metastases and prostate cancer with left iliac lymph node and multiple bone metastases. Interestingly, our imaging revealed different patterns of tumor uptake detected on ^18^ F-FDG and ^18^ F-PSMA-1007 PET/CT in primary lung cancer and lymph node metastases. The primary lung lesion showed intense FDG uptake, and mild uptake with ^18^ F-PSMA-1007. Whereas the mediastinal lymph node metastases showed both intense FDG and PSMA uptake. The prostate lesion, left iliac lymph node, and multiple bone lesions showed significant PSMA uptake and negative FDG uptake.

**Conclusion:**

In this case, there was a homogeneity of ^18^ F-FDG intense uptake between LC and metastatic lymph nodes, but a heterogeneity in ^18^ F-PSMA-1007 uptake. It illustrated that these molecular probes reflect the diversity of tumor microenvironments, which may help us understand the differences of the tumor response to treatment.

## Background

^18^ F-fluorodeoxyglucose positron-emission tomography-computed tomography (^18^ F-FDG PET/CT) has been widely adopted in many international guidelines as a non-invasive technique for primary lung cancer (LC) diagnosis and staging [1–3]. Prostate-Specific Membrane Antigen (PSMA) showed significantly elevated expression levels in prostate cancer (PC) [4]. However, PSMA expression in LC has also been reported in some cases [5]. In the present study, we experienced an interesting case of LC, in which FDG uptake was both intense in primary tumor and metastatic lymph nodes, but PSMA uptake was quite different.

## Case presentation

**Fig. 1:**
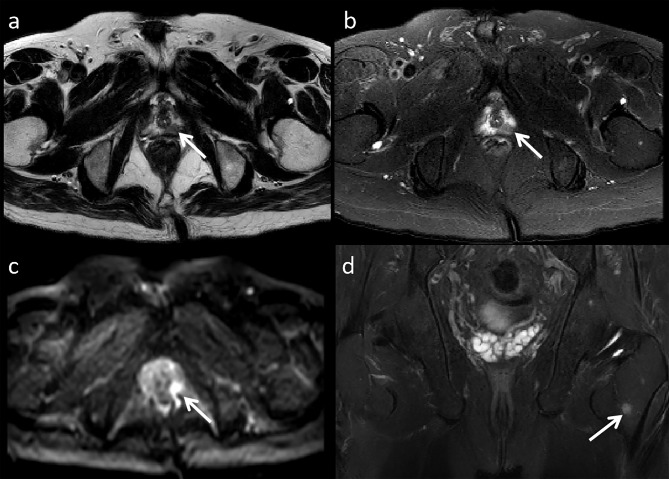
MRI examination of the pelvic cavity. a T2WI showed a hypointense nodule in the left peripheral zone of the prostate. b Axial fat suppressed T2-WI showed a low signal nodule in the prostate. c High b-value DWI map showed a focal area of diffusion restriction in the the prostate. d Coronal fat suppressed T2-WI showed high signal in left upper femur

**Fig. 2:**
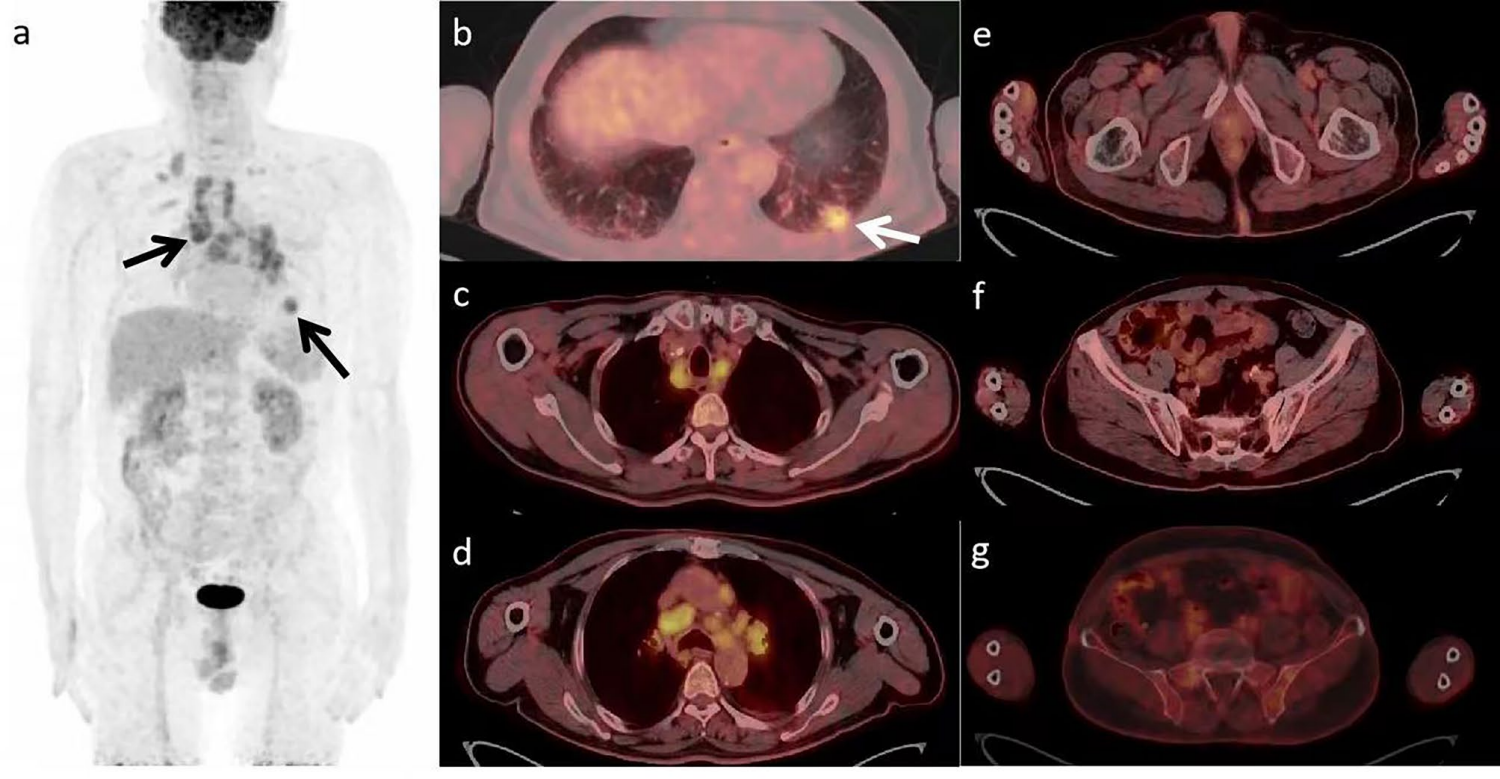
^18^ F-FDG PET/CT images. a Maximum intensity projection (MIP) image showed a increased FDG uptake in left pulmonary nodule and multiple mediastinal metastatic lymph nodes (arrows). b Axial PET/CT fused image showed a hypermetabolic left pulmonary nodule, about 2.4 × 1.9 cm, Maximum Standardized Uptake Value (SUVmax) 5.7 (arrow). c,d Hypermetabolic multiple mediastinal metastatic lymph nodes, SUVmax 4.5-7.0. e,f,g There were no obvious hypermetabolic foci in prostate, pelvic lymph node and pelvic bone

**Fig. 3:**
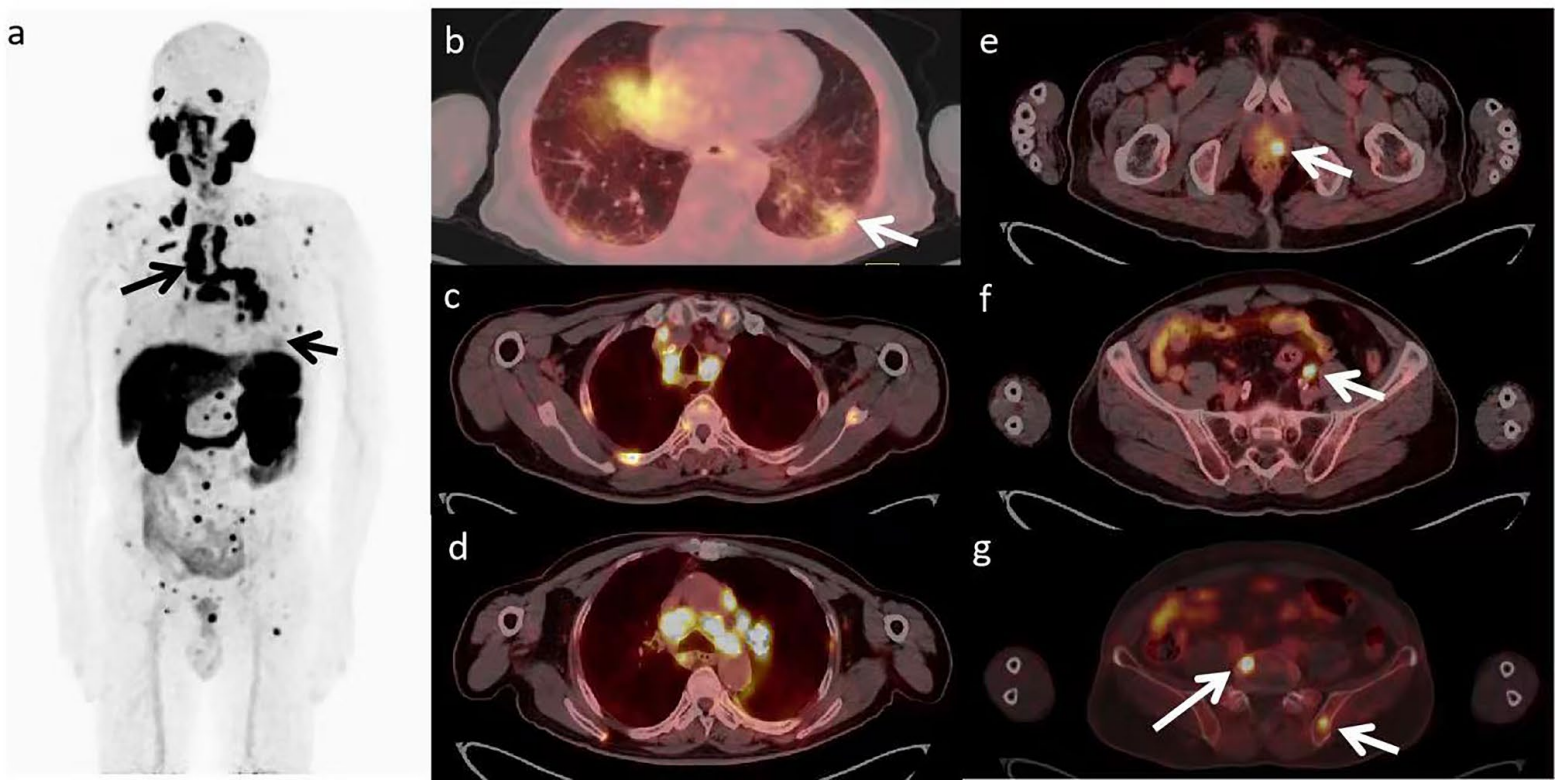
^18^ F-PSMA-1007 PET/CT images. a MIP image showed a increased PSMA uptake in left lobe nodule of prostate, left iliac lymph node and bone lesion. Meanwhile, the left pulmonary nodule and mediastinal lymph nodes had different degrees of PSMA uptake (arrows). b Axial PET/CT fused image showed relatively lower metabolism in left pulmonary nodule, SUVmax 5.1 (arrow). c,d Hypermetabolic multiple mediastinal metastatic lymph nodes, SUVmax 13.2–17.4. e Hypermetabolic nodule in left lobe of prostate, SUVmax 19.1 (arrow). f Hypermetabolic left iliac lymph node, SUVmax 18.5 (arrow). g Hypermetabolic metastasis in L5 vertebra and pelvic bone (arrows), SUVmax 17.0-36.9

A 70-year-old male was admitted to our hospital with a history of intermittent cough for 2 years and recurrent wheezing for 17 months. The blood test showed that Serum-CYFRA211 (10.4ng/ml), SCCA (5.7ng/ml) and PSA (4.18ng/ml) were slightly elevated. The chest CT scan revealed a left pulmonary nodule and enlarged mediastinal lymph nodes. Prostate Magnetic Resonance Imaging (MRI) showed signal abnormality of the left periphery in the prostate and the left upper femur (Fig. 1). Because of suspected LC and PC, the patient underwent ^18^ F-FDG PET/CT and ^18^ F-PSMA-1007 PET/CT, the interval time for the two checks was 1 day. ^18^ F-FDG PET/CT images revealed the left pulmonary nodule and enlarged mediastinal lymph nodes with intense uptake (Fig. 2). ^18^ F-PSMA-1007 PET/CT showed prostate lesion, left iliac lymph node and multiple bone disease lesions were intensely uptaked. The left pulmonary nodule with mild uptake. However, mediastinal lymph nodes with intense uptake (Fig. 3). Biopsy of the lung nodule and mediastinal lymph nodes confirmed NSCLC with lymph node metastases. PC with left iliac lymph node and multiple bone metastases were diagnosed based on cytology and imaging.

## Discussion and conclusions

^18^ F-FDG PET/CT, as a morphological and functional radiographic imaging modality, is widely used for the diagnosis and staging of LC [2]. In recent years, many studies have showed that PSMA is highly overexpressed in prostate cancer cells, and PSMA is expressed in other malignancies as well, including LC [5, 6]. Preclinical data suggests that PSMA might be involved in cancer-related angiogenesis by degrading the extracellular matrix and participating in integrin signal transduction [7, 8]. In NSCLC, in vitro PSMA expression have been reported in 64% of squamous cell carcinomas, 71% of large cell carcinomas, and 45% of adenocarcinomas, more abundantly on neovasculature [9]. In small cell lung cancer (SCLC), in vitro PSMA expression is seen in approximately 70% cases, almost exclusively on neovasculature [6].

PSMA uptake in LC has been previously reported with PC patients incidentally. Usmani reported a case of a 73-year-old man of PC with rising PSA levels. ^68^Ga-PSMA PET/CT was performed, which showed a focal lung lesion, SUVmax 5.6, with subsequent histological confirmation of adenocarcinoma of the lung [10]. In other cases, PSMA imaging of recurrent and metastatic lesions of PC patients showed significant uptake, SUVmax 10.4–22.9, while the uptake of solitary lung nodule was mild, SUVmax 4.4–5.6, suggesting inconsistency with prostate lesions. Finally, Histology of tumor biopsy showed a lung primary tumor rather than lung metastasis of PC [11, 12]. In our case, the left pulmonary nodule showed mild uptake, SUVmax 5.5 in ^18^ F-PSMA-1007 PET/CT, while both PC and its metastases showed intense uptake, SUVmax 17.0-36.9. The aforementioned studies showed that SUVmax is generally thought to be higher in PC or its metastases in comparison to other PSMA-avid malignancies.

However, the uptake pattern of lymph node metastases of LC was still unknown. In this patient, FDG uptake of mediastinal lymph nodes of NSCLC was consistent with primary lesion (primary lesion SUVmax 5.7, mediastinal lymph nodes SUVmax 4.5-7.0), but PSMA uptake was quite different (primary lesion SUVmax 5.1, mediastinal lymph nodes SUVmax 13.2–17.4). Only one case had been reported about the lymph node metastases of LC as a SCLC patient. A 59-year-old man with a background of treated PC, was referred for ^68^Ga-PSMA PET/CT for evaluation. In addition to the PSMA uptake in the known prostate malignancy, the study also demonstrated increased PSMA uptake in left lung hilar soft mass, SUVmax 17.4, along with ^68^Ga-PSMA-avid subcarinal lymph node and aortopulmonary window lymph node, SUVmax 11.2–67.8 [13]. This case showed similar characteristics to our case, in which the PSMA uptake in the lymph nodes was higher than primary lesion. Unfortunately, the ^18^ F-FDG PET/CT examination was not performed in this patient. The aforementioned studies of PSMA PET image suggested that the PSMA expression in LC is not completely consistent with metastatic lymph nodes. However, two histological studies with small sample reported that high immunohistochemical staining for PSMA of primary corresponded with metastatic lesions [14, 15], which differs from the PSMA PET images of two patients mentioned above. So, The heterogeneity of PSMA PET/CT imaging in NSCLC and its metastases will need to be addressed with larger sample prospective trials, including PSMA PET scans, histological expressions and follow-up of tumor treament response and prognosis.

In conclusion, there was a homogeneity of ^18^ F-FDG intense uptake between LC and metastatic lymph nodes, but a heterogeneity in ^18^ F-PSMA-1007 uptake. It illustrated that these molecular probes reflect the diversity of tumor microenvironments, which may help us understand the differences of the tumor response to treatment.

## Data Availability

Data sharing is not applicable to this article as no datasets were generated or analysed.

## Abbreviations

^18^F-FDG-PET/CT: ^18^ F-fluorodeoxyglucose positron-emission tomography-computed tomography
PSMA: Prostate-Specific Membrane Antigen
LC: lung cancer
PC: prostate cancer
MIP: Maximum Intensity Projection
SUVmax: Maximum Standardized Uptake Value
MRI: Magnetic Resonance Imaging
T2WI: T2-weighted imaging
DWI: Diffusion Weighted Imaging
NSCLC: non-small cell lung cancer
SCLC: small cell lung cancer
